# Diagnosis of Coronary Heart Diseases Using Gene Expression Profiling; Stable Coronary Artery Disease, Cardiac Ischemia with and without Myocardial Necrosis

**DOI:** 10.1371/journal.pone.0149475

**Published:** 2016-03-01

**Authors:** Nabila Kazmi, Tom R. Gaunt

**Affiliations:** MRC Integrative Epidemiology Unit, School of Social and Community Medicine, University of Bristol, BS8 2BN, Bristol, United Kingdom; Medical University Hamburg, University Heart Center, GERMANY

## Abstract

Cardiovascular disease (including coronary artery disease and myocardial infarction) is one of the leading causes of death in Europe, and is influenced by both environmental and genetic factors. With the recent advances in genomic tools and technologies there is potential to predict and diagnose heart disease using molecular data from analysis of blood cells. We analyzed gene expression data from blood samples taken from normal people (n = 21), non-significant coronary artery disease (n = 93), patients with unstable angina (n = 16), stable coronary artery disease (n = 14) and myocardial infarction (MI; n = 207). We used a feature selection approach to identify a set of gene expression variables which successfully differentiate different cardiovascular diseases. The initial features were discovered by fitting a linear model for each probe set across all arrays of normal individuals and patients with myocardial infarction. Three different feature optimisation algorithms were devised which identified two discriminating sets of genes, one using MI and normal controls (total genes = 6) and another one using MI and unstable angina patients (total genes = 7). In all our classification approaches we used a non-parametric k-nearest neighbour (KNN) classification method (k = 3). The results proved the diagnostic robustness of the final feature sets in discriminating patients with myocardial infarction from healthy controls. Interestingly it also showed efficacy in discriminating myocardial infarction patients from patients with clinical symptoms of cardiac ischemia but no myocardial necrosis or stable coronary artery disease, despite the influence of batch effects and different microarray gene chips and platforms.

## Introduction

Cardiovascular diseases (CVD) annually cause about 17.3 million deaths worldwide and together are the leading cause of mortality globally [1]. An acute myocardial infarction (MI) is a necrosis of myocardial tissue due to reduced blood supply to the heart and causes around 735,000 heart attacks in the US every year [1]. A large number of scientific advances have been made to prevent, diagnose and treat MI but unfortunately it is still a leading cause of worldwide morbidity and mortality.

The current diagnosis of MI is based on clinical symptoms including chest pain and impaired breathing, changes in the pattern of ECG, and a significant rise and subsequent fall in the circulating levels of cardiac troponins (cTns) [2]. Despite the advances in the cardiovascular field there are several limitations in the current diagnostic system. The advances in hs-cTn assays have made it possible to detect 10 fold lower circulating Tn concentrations (increased sensitivity) but have also elevated the number of cardiovascular patients by including clinically non-diseased people showing changes in cTns due to other conditions (decreased specificity) [3]. Another diagnostic measure is the detection of cardiac miRNAs, which are introduced as sensitive biomarkers [4] but their successful detection is inhibited due to their low abundance, small size and tissue specific expression. Their role as biomarkers may become more prominent with the invention of fast standardised and automated detection systems [5]. BNP, CRP and other serum inflammatory markers are also considered as cardiovascular biomarkers but they have only made an incremental improvement to diagnosis [6,7, 8].

The majority of cardiac biomarkers are developed using the knowledge of pathological and physiological processes in established pathways. In contrast microarray platforms measure the expression of a large number of genes simultaneously, enabling gene expression profiling across many pathways in parallel. This approach has the potential to represent a comprehensive range of pathophysiological processes of CVD economically and efficiently [9]. Gene expression profiling extends beyond known biomarkers to reveal potential biomarkers which have not previously been reported as associated with CVD.

Gene expression analysis can enable us to understand and discover novel and sensitive biomarkers of cardiovascular disease. A number of studies have published work in this area: a gene expression analysis yielded 482 genes with an association to the composition of coronary atherosclerotic plaques and most of them were not previously linked to atherosclerosis [10]. A wide-scale gene expression profiling identified fifty six divergent genes for atherosclerotic and non-atherosclerotic human coronary arteries, wherein 49 were never associated with CAD before [11]. Elashoff and co-authors identified a set of classifying genes which with the information of age and sex were strongly correlated to obstructive CAD in non-diabetic patients [12]. The divergent gene expressions were identified which discriminated ischemic and non-ischemic cardiomyopathies conditions among the end-stage patients [13, 14]. In another study microarray analysis and gene expression profiling were used to discover genes related to heart failure using the expression profiles of 12 patients with heart failure [15], another study of normal controls and MI patients discovered genetic markers and dysregulated pathways associated with disease recurrence in first time MI patients [16].

There is a key question of how well blood transcriptome represents transcriptional changes in the heart. Liew *et al* report a genome wide survey using microarrays and expressed sequence tags, in which the peripheral blood transcriptome was compared to the transcriptome of nine different human tissues including heart. This comparison showed an overlap of 80% with any given tissue and 84% with heart, suggesting peripheral blood transcriptome is an inexpensive and readily accessible tool to proxy gene expression in other tissues [17].

Despite a range of studies exploring differential expression in cardiovascular outcomes no attempt to use this information to classify patients according to outcome (eg unstable angina and MI) has been reported yet. If successful this approach offers the potential to provide a diagnostic tool to sub-classify patients. In this work, we identified the discriminatory features to differentiate among normal people (with normal cardiac function), patients with MI, stable coronary artery disease (CAD) and unstable angina using gene expression in blood cells. Blood transcriptome was used as this is an easily accessible tissue for diagnostic purposes. This paper also discusses the success and classification accuracy of different proposed algorithms implemented to discover the potential divergent gene expression features of heart diseases and their optimisation to explore the subset of most discriminatory features.

## Materials and Methods

### Incorporated Datasets

#### The “Nelson Dataset”

This comprises gene expression data from blood samples taken from 47 subjects, including 26 first time acute MI patients (within 48-hours post-MI) and 21 normal controls. Normal controls were enrolled at the Mayo Clinic Rochester echocardiography laboratory and had a normal cardiac function with no history of previous cardiac diseases. The controls and MI patients were matched by sex and age [16].

The dataset was randomly subdivided into 2 datasets NelsonA (n = 30; 15 first time MI and 15 controls) and NelsonB (n = 17; 11 first time MI and 6 controls) due to limited sample numbers. NelsonA was used as a training dataset to build the classifier and NelsonB was used to select the final classifier and for the independent validation. The accession number of the dataset at NCBI Gene Expression Omnibus (GEO) is GSE48060.

#### The “Rothman Dataset”

The mRNA expression levels from whole blood of 26 patients with acute coronary syndrome (ACS) were taken at 7 and 30 days post ACS. The dataset has 52 ACS samples including eight patients with unstable angina and eighteen with myocardial infarction (MI) (GEO; GSE29111). The Rothman Dataset has samples collected at two different time points therefore we divided it into two subsets; Rothman-Timepoint1 (samples taken at 7^th^ day) and Rothman-Timepoint2 (samples taken at 30^th^ day).

Both the Nelson and Rothman datasets were processed on the Affymetrix Human Genome U133 plus 2.0 platform and were normalised using the robust multi-array averaging (RMA) method of the “affy” package in R [18].

#### The “Beata Dataset”

Blood samples were collected from twenty eight patients with ST-segment elevation myocardial infarction (STEMI) and fourteen controls (who were patients with stable coronary artery disease, CAD) [19]. The control samples were categorised into patients with coronary angiography (at least one stenosis exceeding 50% or previous coronary angioplasty or previous coronary artery bypass graft), or with non-invasive tests (positive exercise test) and showed no symptoms of MI. The samples of STEMI patients were taken on the first day, after four-six days and after six months from their admission date, giving a total of 98 samples. All samples were assayed on the Affymetrix GeneChip Human Gene 1.0 ST (GEO; GSE62646).

STEMI samples were categorised into three sets based on their collection time points; Beata-Admission, Beata-Discharge and Beata-6Months. These three subsets were analysed with the same control samples.

#### The “Gregg Dataset”

A research group collected blood samples of 338 subjects with mixed cardiovascular phenotypes, and either confirmed or suspected CAD, undergoing cardiac catheterization [20]. In our study, we included patients with non-significant CAD (controls; n = 93) and first time MI patients (n = 61) among 338 subjects. Subjects were diagnosed with non-significant CAD if the visible plaque resulted in <50% luminal stenosis and MI was diagnosed using standard universal criteria. RNA expression levels were measured on Illumina HumanHT-12 V4.0 expression beadchip (GEO; GSE49925).

The structure of datasets, their subsets and utilisation is summarised in S1 Fig and S2 Fig. These figures describe total number of samples, number of cases and controls, datasets split based on different time points, training and test sets and utilisation of each dataset.

In order to carry out the quality analysis on each dataset, boxplots were created to plot the samples non-normalised gene expression distribution on each chip. On visualisation of these plots (S3 Fig), we did not identify any problematic chips (i.e. outliers which were substantially different from the distribution of other chips in the same dataset). We observed higher variability in the expression measures of Gregg dataset but no array was identified as a potential outlier. We also created boxplots after normalising each dataset, normalisation greatly reduced the variability and post-normalisation samples looked very similar within each dataset (S4 Fig).

All included studies are publically available and are covered by their own ethical approvals. The ethics statements of these studies can be found in their published articles [16, 19, 20].

### Classifier Development

**Fig 1:**
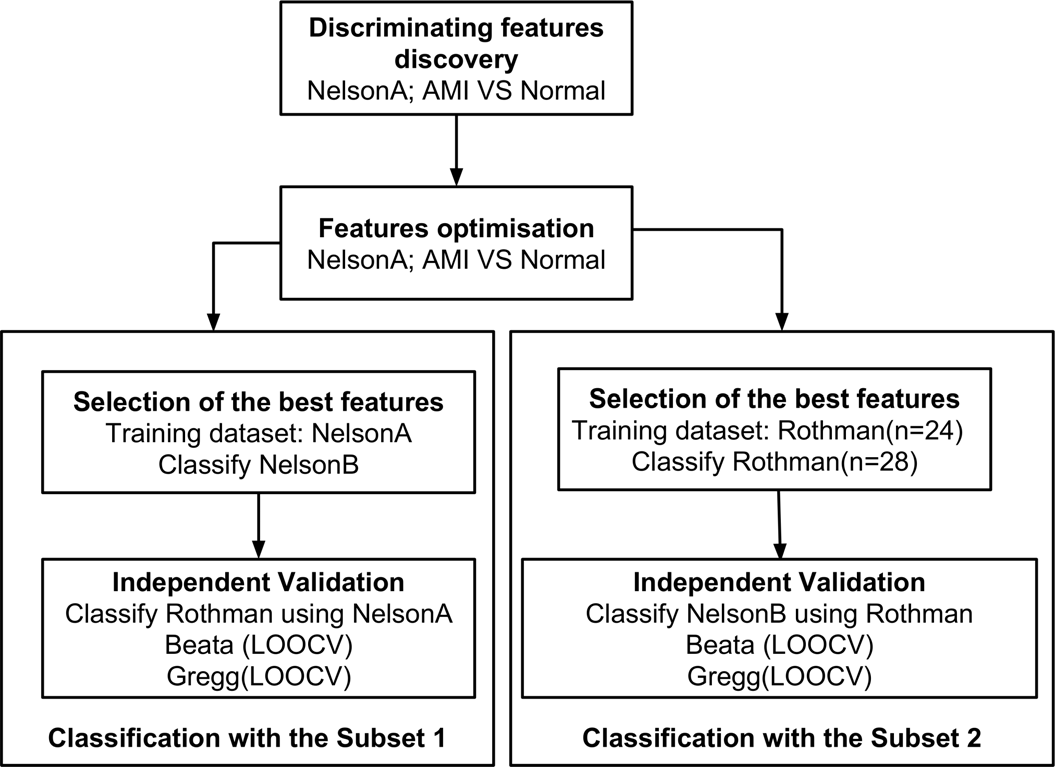
A flow chart to describe the classification process.

The expression measures of NelsonA were used to extract the initial differential features and to build the classifier. We used NelsonA to optimise the initial set of features in three different optimisation techniques and generated several subsets. We then used Nelson (NelsonA as a training set and NelsonB a test set) and Rothman was randomly split into training and test sets (n = 24, training set (12 patients with unstable angina as controls and 12 MI patients) and n = 28 test set (4 controls and 24 MI patients)) to select the final features, Subset 1 and Subset 2 respectively (based on their highest classification accuracy). This process (random sampling to produce training (n = 24) and test (n = 28) subsets) was repeated 15 times to ensure that the achieved performance was not due to the sampling effects. We included all samples from the Rothman dataset (rather than using Rothman-Timepoint1 or Rothman-Timepoint2 subsets) as the classifier selection approach requires data split into two almost equal sized subsets (training and test sets; Fig 1). If we consider either Rothman-Timepoint1 or Rothman-Timepoint2 there is an imbalance in the proportion of cases and controls in the training set.

Both subsets, Subset 1 and Subset 2, were independently validated on the Rothman, Beata and Gregg datasets using two different approaches: blind validation and leave one out cross validation (LOOCV). In all our classification approaches, we applied a non-parametric k-nearest neighbour (KNN) classification method (k = 3) which is simple to understand and does not make any assumptions about the data distributions [21–23]. Our prediction models provided the training examples and their classes to a KNN algorithm which then predicted the class of each test sample using the expression measures of only those probe sets which were in the training set. A flow chart explaining the discovery and validation process of these two classifiers is given in Fig 1.

**Fig 2:**
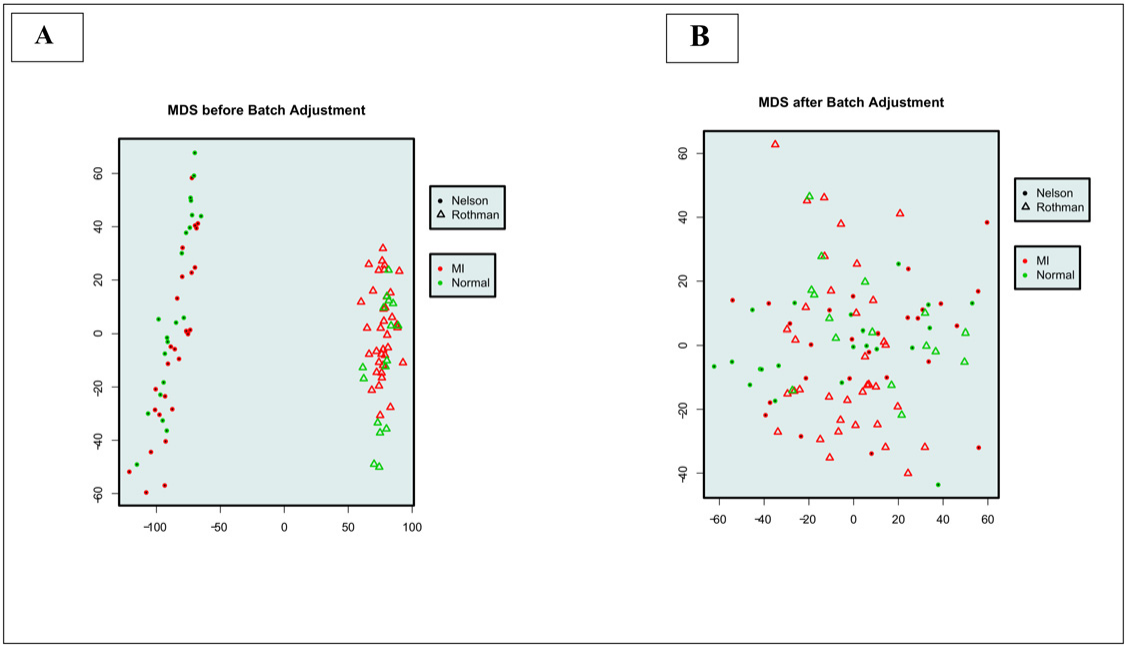
Batch effects and their COMBAT adjustment on merging Nelson and Rothman datasets.

Subset 1 and the Subset 2 were not validated on Nelson and Rothman respectively because these datasets were used to select the final classifiers. In blind validation of both the Rothman dataset (with Subset 1 using NelsonA as a training set) and the NelsonB dataset (with Subset 2 using Rothman as a training set), we combined two different datasets, and this merge was expected to have some non-biological experimental variation. We created Multidimensional Scaling (MDS) plots to trace any expected batch effects (Fig 2A) and used a parametric and nonparametric empirical Bayes framework (COMBAT) to adjust for them (Fig 2B) [24]. We adjusted for batch effects in the blind validation step only because here we combined two different datasets while in LOOCV we used a single dataset.

Though the Beata dataset has STEMI and CAD samples we still classified it using the features that were originally discovered using MI and normal controls. The reasons to consider Beata were: (a) limited relevant public datasets, (b) myocardial infarction occurs in STEMI patients and (c) to test the robustness and accuracy of the classifier across different closely associated diseases (i.e. generalizability).

We compared our classifier’s performance with a well-known machine learning algorithm, Random forests. We used a package *randomForest*, which is developed in R, and implements Breiman’s Random forests algorithm [25]. The Random forests classifier was trained using all genes in the NelsonA dataset where the number of variables available per split at each tree node was the square root of the number of predictor variables and the number of trees was 10001.

#### Feature Selection (Discovery Phase)

Initial selection of the most discriminating features was performed using two different approaches, which are described below.

1. P-Value based Selection (Discovery Method 1): A linear model (lmFit) was fitted to training samples and then an empirical Bayes method was used to compute the p-values corresponding to the t-statistics of differential expression [26]. The probe sets (PS) were ranked according to their most significant p-values, which were adjusted by using Benjamini and Hochberg (BH) method. The BH method controls the expected false discovery rate (FDR) below a specified threshold and is considered an appropriate choice for microarray experiments [27]. The PS were retained with a BH-adjusted p-value < 0.05 and this criteria selected 636 PS for the initial classifier.
2. LOOCV t-tests (Discovery Method 2): A linear model (lmFit) was fitted in a LOOCV manner to all training samples [28]. LOOCV left one sample out and identified features with a BH adjusted p-value < 0.05 using the remaining samples. This leave one out process was repeated for all samples. Finally, the most discriminatory genes were retrieved by their maximum number of appearances in the top ranked genes of LOOCV t-tests. This method gave 2727 genes but we selected the top 636 to give a comparable number of features to those identified using Discovery Method 1.

#### Features Optimisation

Microarray gene expression profiling offers many advantages to the research community including measuring a large number of features at a time, but control for false positive rate is challenging. We developed three different optimisation algorithms to identify the most discriminatory features among two basic lists identified in Discovery Method 1 and Discovery Method 2. These novel feature selection methods will maximize the true positives and provide a control over false positives. All the following methods processed both lists individually. The NelsonA dataset was used to optimise the lists.

1 Optimisation on Success Rate [Optimisation 1]: Initiated with the expression measures of the top two PS (among the list generated in Discovery Method 1) and tested on the NelsonA samples in a LOOCV manner (LOOCV: classified one sample using remaining samples in the training set). The LOOCV process was repeated until all samples were classified using the selected PS. Then on the next iteration, the next PS of the list was included and tested using LOOCV again. This whole process was repeated until all PS were included. After processing of each PS, we recorded the classifiers success rate (SR or classification accuracy is the percentage of correct classifications) and ΔSR (SR_NEW_—SR_PREVIOUS_). PS which impaired the performance with a negative ΔSR were eliminated from the list and were given no opportunity for any further LOOCV.

Exactly the same procedure was adopted to optimise the classifying list identified in Discovery Method 2. The optimisation of features discovered in Method 1 reduced 636 PS to 22 and in Method 2 reduced 636 PS to 64.

2 Performance Improvement and Random Crossover [Optimisation 2]: The objective of this and the next optimisation technique was to reduce the initial classifying lists to an optimised set using evolutionary algorithms. The method divided all 636 PS into fixed size subsets. For each subset, LOOCV was performed on NelsonA dataset with 1–2,…, n PS of the subset and then the SR and ΔSR was calculated for each PS in the same manner as was calculated in Optimisation Method 1. Any PS which impaired the performance was eliminated from the list used for the cross validations but kept its place in the original subset for evaluation after the crossovers. After processing of the subset, all PS were ranked according to their ΔSR and the top 25 were retained to perform LOOCV and recorded as an optimised subset on showing a satisfactory SR. Our feature selection methods; Optimisation 2 and Optimisation 3 reduced initially discovered 636 PS into 25 most informative PS. The aim was to identify and drop the bulk of irrelevant and redundant attributes from data (which add noise and compromise performance and accuracy of the models) prior to performing feature selection. We considered 25 as a sensible number because we anticipated the length of the final classifiers (after feature selection) will be much shorter than this number.

The process was evolved for a predefined number of iterations to discover the best combinations.

**Crossover**: 2 point crossover was performed between adjacent subsets. After all crossovers the new subsets were evaluated using the cross validation.

3 Performance Improvement and Crossover of the Fittest [Optimisation3]: This method works in a similar pattern as Optimisation 2 and differs only in the crossover technique. **Crossover**: Each subset ranked its PS according to the ΔSR and performed crossover of its PS with best performing PS of other subsets. After completing all crossovers the newly evolved subsets were evaluated using cross validation.

The pseudo-code of these three optimisation methods are provided in S1 File.

## Results

### Selection of the Final Classifiers

The subjects of the NelsonA dataset were used as a standard training set to classify NelsonB in order to identify the final classifier among all optimised lists. We identified a set of seven PS which were originally identified using Optimisation 2 on the features of Discovery Method 2 and resulted in 100% SR. Here we call this list **Subset 1**. Another subset of nine PS (**Subset 2**; Optimisation 2 and Features Discovery Method 2) was identified among the optimised lists after the classification of Rothman. Subset 2 classified MI patients from patients with unstable angina with the highest SR = 88%. The SR was also calculated for the original lists of both Subset 1 and Subset 2; Optimisation 2 on the features of Discovery Method 2 and Optimisation 2 and Features Discovery Method 2 respectively (each of length 25 PS) and is plotted in S5 Fig.

A performance comparison of several optimised lists is given in Table 1; these measures were used to select the final classifiers. The distribution of each identified PS of both classifiers in the original training data (NelsonA) is provided in S6 Fig and S7 Fig. We compared the very initial classifying PS lists with the 157 probe sets identified in the Nelson *et al* original publication [16] and found an overlap of 100 and 103 PS with our 636 PS from Discovery Method 1 and 636 PS from Discovery Method 2 respectively. Comparison with the final classifiers returned 2 and 4 overlapping PS for Subset 1 and Subset 2 respectively. The PS identified in Nelson’s work were not completely matched to our identified lists possibly because they identified the differential features with FDR control at 0.05 and fold change of 1.2 from acute MI patients and control subjects.

**Table 1:** The SR of the optimised feature lists on the NelsonB and Rothman datasets.

Dataset	Opt1D1	Opt1D2	Opt2D1	Opt2D2	Opt3D1	Opt3D2
NelsonB	53%	65%	95%	**100%**	95%	95%
Rothman	63%	70%	87%	**88%**	86%	87%

This table shows the classification performance of six optimised lists (optimised using NelsonA) on Nelson and Rothman datasets. Opt1D1 indicates reduced features set after the Optimisation Method 1 on Feature Discovery Method 1, Opt2 = Optimisation Method 2, Opt3 = Optimisation Method 3, D2 = Feature Discovery Method 2.

### Independent Validation

Subset 1 was used to classify the independent dataset Rothman using the NelsonA dataset as a standard training set (Blind Validation). Subset 1 resulted in 81% SR and 66% SR for Rothman-Timepoint1 and Rothman-Timepoint2 respectively. Then Subset 2 was used to classify NelsonB using Rothman-Timepoint1 and Rothman-Timepoint2 as individual training sets (Blind Validation). The SR was 59% and 65% respectively.

For the classification of the Beata dataset (n = 98), we mapped our classifying probe sets to the HuGene-1_0-st platform using BioMart (http://www.biomart.org/). Biomart used the probe set identifier to map them across different platforms. Unfortunately, we could not map all probe sets of the final classifiers; 5/7 PS were mapped for Subset 1, with an additional one PS missing in the Beata dataset, giving 4 in total. For Subset 2, 2/8 PS were mapped (both were available in the dataset). The gene expression data were normalised using the RMA method. We labelled CAD patients as controls and STEMI patients as cases, performed 15 times random sampling and classified all samples in a LOOCV manner. Random sampling was used to keep the groups balanced for the KNN algorithm to avoid any sampling effects. Sampling divided each subset into 14 MI cases and 14 controls. LOOCV left one Beata sample out as a test sample and used only PS of Subset1 or Subset2 from the training dataset (n-1 samples) and then classified the held out sample and recorded the classification results. The process was repeated until each sample had been held out and classified using the samples in the training set. Finally, LOOCV provided an average success rate, sensitivity and other classification measures to judge the performance of the classifiers.

For the classification of the Gregg dataset, all classifying probe sets of both Subset 1 and Subset 2 were mapped from Affymetrix Human Genome U133 plus 2.0 to Illumina HumanHT-12 V4.0 expression beadchip platform using BioMart. For Subset 1, we mapped 4/7 PS and for Subset 2 5/8 PS. The average signal intensities of the dataset were log_2_ transformed then normalised using quantile normalisation [29]. 14,343 probes among 47,211 were constantly detected above the background. The subjects were recruited in two phases and 14,111 probe sets were common in both phases. Unfortunately, 2 among 4 mapped PS of Subset 1 and one among 5 mapped probe sets of Subset 2 were among the 232 missing probes. LOOCV was performed using Subset 1 and Subset 2 and classified 154 patients after performing fifteen random samplings. The recorded average SR was 49% for 2 mapped PS of Subset 1 and 51% for the 4 mapped PS of Subset 2.

The classification statistics including SR, sensitivity, specificity, positive predictive value (PPV), negative predictive value (NPV) are given in Table 2. The upper limit of 95% confidence interval (CI Upper) and lower limit of 95% confidence interval (CI Lower) for classification accuracy, sensitivity, specificity, PPV and NPV are given in Table 3. We used the LOOCV approach therefore in calculating the confidence interval, we took the classification results in each fold of LOOCV (which is either 100% or 0% for each example) and then calculated the standard deviation across these values.

**Table 2:** The classification statistics, including SR, sensitivity, specificity, positive predictive value (PPV) and negative predictive value (NPV) were recorded after the classification of each dataset.

Dataset	SR	Sensitivity	Specificity	PPV	NPV
NelsonB1	59%	0.82	0.17	0.64	0.33
NelsonB2	65%	0.73	0.5	0.73	0.5
Rothman1	81%	0.78	0.88	0.93	0.64
Rothman2	66%	0.72	0.5	0.76	0.44
Beata-A-1	76%	0.69	0.83	0.8	0.73
Beata-A-2	82%	0.8	0.83	0.83	0.8
Beata-D-1	65%	0.62	0.67	0.66	0.65
Beata-D-2	78%	0.75	0.79	0.78	0.77
Beata-6M-1	59%	0.6	0.56	0.59	0.58
Beata-6M-2	66%	0.64	0.67	0.67	0.65

**Table 3:** Upper limit of 95% confidence interval (CI Upper) and lower limit of 95% confidence interval (CI Lower) for SR, sensitivity, specificity, PPV and NPV were recorded after the classification of each dataset.

Dataset	CI (SR)	CI (Sensitivity)	CI (Specificity)	CI (PPV)	CI (NPV)
NelsonB1	35–83	60–100	-16-50	38–90	-32-10
NelsonB2	41–90	045–100	6–93	45–100	6–94
Rothman1	65–96	60–97	63–100	80–100	34–93
Rothman2	47–84	51–93	13–87	55–97	10–80
Beata-A-1	60–92	45–93	65–100	61–100	50–95
Beata-A-2	67–96	58–100	63–100	62–100	59–100
Beata-D-1	47–83	39–90	40–89	39–90	40–89
Beata-D-2	61–93	50–97	61–100	58–100	54–98
Beata-6M-1	40–77	35–87	32–82	35–85	32–85
Beata-6M-1	48–83	37–88	44–94	40–93	41–90

**Fig 3:**
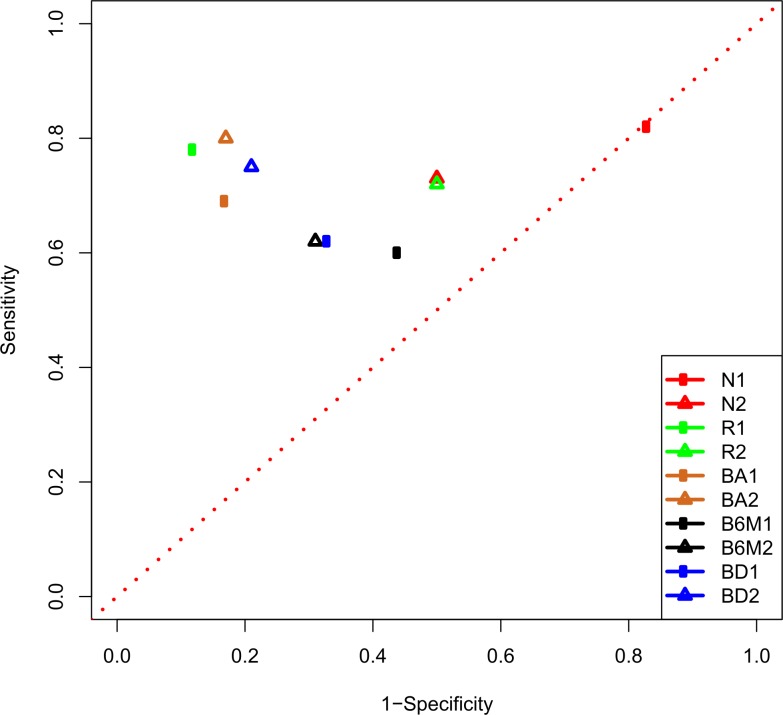
The ROC graph is plotted to show the performance of the binary classifiers.

A receiver operating characteristics (ROC) graph was plotted to measure the quality of both discrete binary classifiers Subset 1 and Subset 2; (Fig 3). The ROC analysis categorised MI patients as positive case and non-MI patients as controls.

Subset 2 was used to classify the NelsonB dataset using both the expression measures of Rothman-Timepoint1 (N1) and Rothman-Timepoint2 (N2). Subset 1 classified Rothman-Timepoint1 (R1) and Rothman-Timepoint2 (R2) using the expression measures of NelsonA. BA1 = Subset 1 on Beata-Admission, BA2 = Subset 2 on Beata-Admission, BD1 = Subset 1 on Beata-Discharge, BD2 = Subset 2 on Beata-Discharge, B6M1 = Subset 1 on Beata-6Months, B6M2 = Subset 2 on Beata-6Months.

In a comparison of our classifiers and Random forests, the cross validation of Random forests classifiers on NelsonB which was trained on NelsonA showed 59% SR which is substantially weaker than our classifier’s performances (100% SR of the Subset 1 on NelsonB using NelsonA as a training set). Random forests calculated an importance measure for each variable (PS) using Gini criteria (mean decrease in Gini index). We identified the top 636 PS among the most important PS identified by Random forests and compared them with our initial classifiers. We found an overlap of 336 (of 636) and 333 (of 636) PS with PS Discovery Method 1 and PS Discovery Method 2 respectively.

## Discussion

We used the NelsonA dataset to identify maximally discriminating features between normal controls and MI patients using two different methods; Discovery Method1 and Discovery Method 2. We generated two separate feature lists where 611 probe sets were overlapping but were at different positions. We then applied three different optimisation techniques on both feature lists using NelsonA dataset and generated six different optimised feature lists. Then, we used Nelson and Rothman datasets to select our final classifiers using highest classification accuracy as our selection criterion.

The final selected classifiers were independently validated on the Nelson, Rothman, Beata and Gregg datasets following two procedures: LOOCV and blind validation. We used blind validation where possible to measure the robustness of a classifier as the test dataset is entirely “blind to” (independent of) the training set. Therefore, we classified NelsonB using Rothman and Rothman using NelsonA in a blind manner. For Beata and Gregg datasets, we were forced to use LOOCV because there was no suitable independent training set on the same transcriptomic platform.

ROC analysis was carried out to prove the diagnostic potential of our binary classifiers (Fig 3). We plotted a ROC graph (not a ROC curve) because our classifiers were discrete binary classifiers not scoring classifiers [30]. In independent validation, Subset 1 classified Rothman-Timepoint1 and Rothman-Timepoint2 (n = 28) using the expression measures of NelsonA (R1and R2; Fig 3). The Rothman dataset is a population of patients with unstable angina and MI and we used the expression measures of NelsonA (for training), which has fifteen normal controls as negative cases and fifteen MI patients as positive cases. The ROC graph showed the classifier’s performance (R1; Fig 3) near *the perfect classification point* (1 [sensitivity], 0 [1-specificity]) with 0.78 sensitivity and 0.88 specificity. High sensitivity and specificity values indicate that the classifier has a high measure of completeness and exactness and is quite robust in predicting MI from unstable angina even under the influence of batch effects. The classification success for Rothman-Timepoint2 was slightly compromised as compared to samples of time point1. The Rothman dataset is not comprehensively documented, and in our opinion external factors (eg treatment/medication) might have slightly altered the gene expression profiles of some of the MI patients, lowering the sensitivity to 0.72. Alternatively, the increased time might have resulted in some of the patients with unstable angina progressing to MI with a weakened specificity (0.5) (S8 Fig).

Subset 2 was used to classify the NelsonB dataset using both the expression measures of Rothman-Timepoint1 (N1; Fig 3) and Rothman-Timepoint2 (N2; Fig 3) with sensitivity = 0.82 and 0.73 respectively. Using the expression measures of unstable angina (negative cases) and MI patients (positive cases), Subset 2 discriminated positive cases (MI patients; NeslonB) very well with a high sensitivity. The observed FPR was also very high. There is a possibility that batch effects, the negative cases of the training dataset which were samples of another cardiac ischemia and small training set (n = 16; eight controls and eight cases) might have their influence. An improved specificity and reduced sensitivity was observed when we changed the training dataset from Rothman-Timepoint1 to Rothman-Timepoint2, which might indicate that some patients of unstable angina improved their cardiac health and (in terms of expression) became more like normal controls whilst the rest of them moved towards MI.

**Fig 4:**
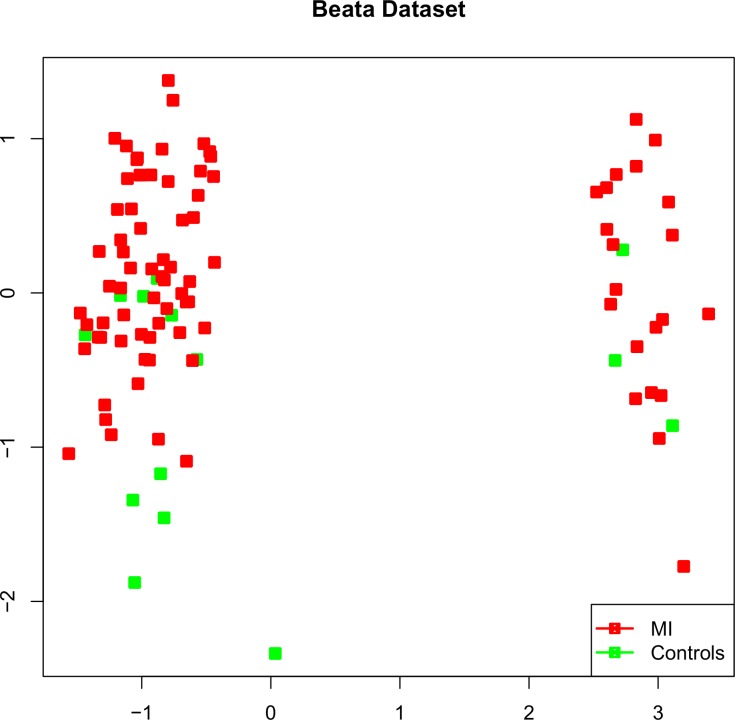
A MDS plot created for Beata dataset to show the distribution of its cases and controls.

The Beata dataset contains a sample of STEMI and CAD patients and was classified in a LOOCV manner, where both the training and test samples were from the same dataset. For classification of this dataset Subset 2 (selected using the information of two diseases; MI and unstable angina) showed higher classification performance compared to Subset 1 (identified using MI and normal controls), suggesting Subset 2 was more appropriate for discriminating the diseases (BA1 = Subset 1 on Beata-Admission, BA2 = Subset 2 on Beata-Admission, BD1 = Subset 1 on Beata-Discharge, BD2 = Subset 2 on Beata-Discharge, B6M1 = Subset 1 on Beata-6Months, B6M2 = Subset 2 on Beata-6Months; Fig 3). For a small number of classifying PS (2 and 4), we recorded a fairly high classification success rate. To rule out any possibility of batch effects separating the controls and cases, we generated a MDS plot using the 100 least differentially expressed features. These 100 Affymetrix Human Genome U133 plus 2.0 PS were mapped to 62 Affymetrix GeneChip Human Gene 1.0 ST PS using Biomart. 42 among 62 mapped PS were found in the Beata dataset. The MDS plot showed a mixed distribution of controls and cases reflecting the power of our classifiers (Fig 4). The classification success decreased with time elapsed from diagnosis/admission time (as we observed in the Rothman dataset). The SR was the highest at the time of admission, slightly decreased at the time of discharge after 3–4 days of the diagnosis and was lowest after six months. These statistics might indicate that environmental factors (e.g. treatment) reduce the difference in gene expression profiles between cases and controls from what they were at the time of diagnosis.

For the Gregg dataset classification, we could not map many PS for both classifiers and got a very low SR. The reasons may include a small number of mapped PS and the use of a completely different platform as compared to the other three datasets.

### Genes in the classifiers

#### Subset 1

*RHOBTB3* (Rho-related BTB domain-containing protein 3) is involved in transport of vesicle docking at the Golgi complex, possibly by participating in releasing *M6PRBP1*/TIP47 from vesicles to permit their efficient docking and fusion at the Golgi [31]. Elastin Microfibril Interfacer 2 (*EMILIN2*) is a protein-coding gene and is associated with porokeratosis of mibelli and porokeratosis diseases. It has cell adhesive capability and may be responsible for connecting smooth muscle cells with elastic fibers and may also regulate vessel assembly [32]. *PARP6* (Poly (ADP-Ribose) Polymerase Family, Member 6) is a protein-coding gene and is associated with diphtheria. Poly (ADP-ribose) polymerase (PARP) adds multiple ADP-ribose moieties and activates the post-translational modification of proteins. PARP inhibitors are being developed for use in cancer, diabetes, stroke and cardiovascular disease [33] (http://www.genecards.org/cgi-bin/carddisp.pl?gene=PARP6).

RNA binding protein 5 (*RBM5*) is a candidate tumor suppressor gene and encodes a nuclear RNA binding protein. The encoded protein is involved in the onset of cell cycle arrest and apoptosis through pre-mRNA splicing of multiple target genes e.g. the tumor suppressor protein *p53* [34, 35]. *MT-TE* (Mitochondrially Encoded TRNA Glutamic Acid) is a RNA gene and is associated with mitochondrial myopathy with reversible cytochrome c oxidase deficiency and mitochondrial myopathy with diabetes [36]. *MT-ND6* (Mitochondrially Encoded NADH Dehydrogenase 6) is a protein-coding gene and is associated with melas syndrome and leber hereditary optic neuropathy with dystonia (http://www.genecards.org/cgi-bin/carddisp.pl?gene=MT-ND6).

#### Subset 2

CH507-513H4.3, CH507-513H4.6, CH507-513H4.5 and CH507-513H4.4 are large intergenic non-coding RNAs (lincRNAs). lincRNAs are emerging as the main regulators of several cellular processes(http://vega.sanger.ac.uk/Homo_sapiens/Gene/Summary?g=OTTHUMG00000189716;r=21:8210384-8211306;t=OTTHUMT00000481341). Calponin 2 (CNN2) is a protein that may have an important role in cell adhesion and smooth muscle contraction [37]. It can bind to calmodulin, actin, troponin C and tropomyosin. Its function may also include the structural organization of actin filaments [38]. Exosome Component 1 (*EXOSC1*) encodes a core component of the exosome. The mammalian exosome plays a key role in rapid degradation of AU rich element-containing RNAs but not in poly (A) shortening. The protein is associated with the exosome by protein-protein interactions with ribosomal RNA-processing protein 42 and ribosomal RNA-processing protein 46 [39, 40]. Solute Carrier Family 19 (Folate Transporter), Member 1 (*SLC19A1*), encodes membrane protein and is known as a transporter of folate and plays a part in the regulation of intracellular concentrations of folate [41].

### Study Limitations

Although the classifiers sensitivity measures were good throughout the analysis we could not fully justify the specificity measures for some experiments due to a limited number of samples.

## Conclusions

Using gene expression analysis, we identified two sets of genes which showed a promising performance in classifying groups of healthy people, patients with stable CAD, unstable angina and MI. Our results support the utilization of the discovered genes and proposed methods in the diagnosis of cardiovascular diseases using peripheral blood gene expression and suggest potential clinical applications of gene expression data as biomarkers in cardiovascular disease.

## Supporting Information

S1 Fig A diagrammatical description of Nelson and Rothman datasets detailing controls and cases, any data split and their usage in independent validation. (TIF)

S2 Fig A diagrammatical description of Beata and Gregg datasets detailing controls and cases, any data split and their usage in independent validation. (TIF)

S3 Fig Quality check, boxplots were created using pre-normalised gene expressions for four included datasets. (TIF)

S4 Fig Quality check, boxplots were created using post-normalised gene expressions for four included datasets. (TIFF)

S5 Fig The success rate of two optimised lists each length of 25 PS which were then reduced to select the Subset1 and the Subset2. (TIFF)

S6 Fig The distribution of each PS (the Subset 1) in NelsonA dataset. (TIF)

S7 Fig The distribution of each PS (the Subset 2) in NelsonA dataset. (TIF)

S8 Fig A MDS plot was generated using the expression measures of the Subset 1 of Rothman dataset. (TIF)

S1 File The pseudocode of each optimisation algorithm. (PDF)
