# Supplementary figures and images for: Diagnosis of Coronary Heart Diseases Using Gene Expression Profiling; Stable Coronary Artery Disease, Cardiac Ischemia with and without Myocardial Necrosis
Source: PLoS One. 2016 Mar 1;11(3):e0149475. doi: 10.1371/journal.pone.0149475 (PMC4773227; doi:10.1371/journal.pone.0149475)

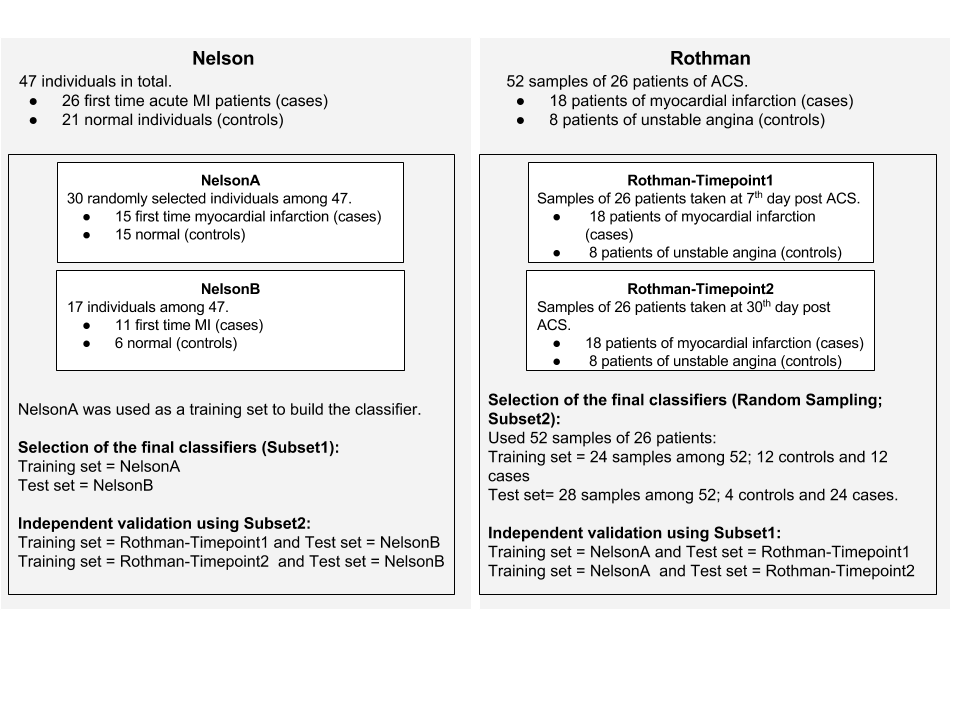

Supplement: S1 Fig — (TIF) [file pone.0149475.s001.tif]

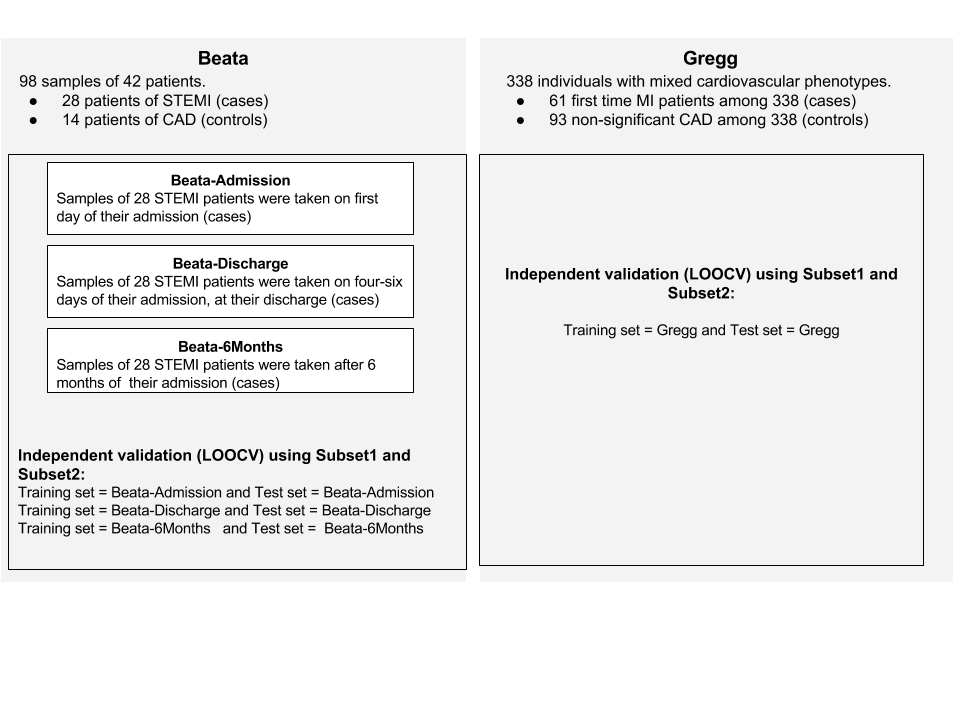

Supplement: S2 Fig — (TIF) [file pone.0149475.s002.tif]

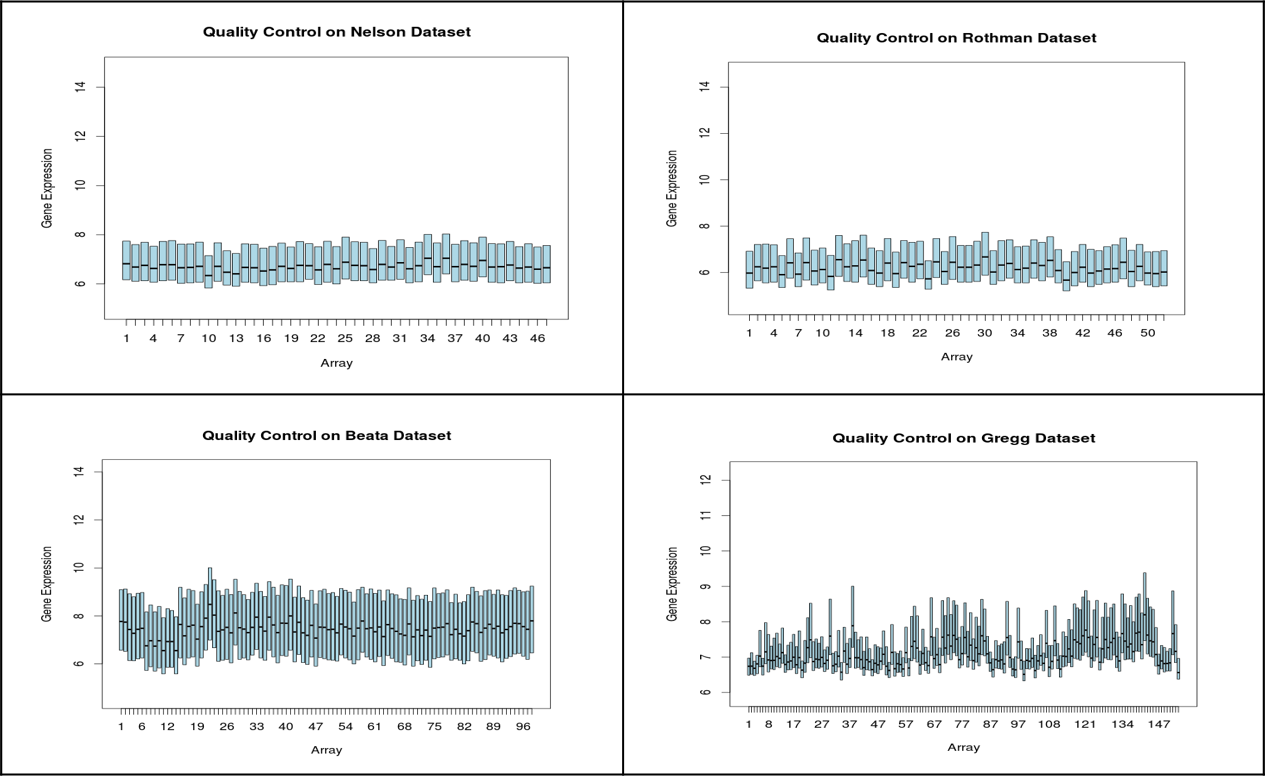

Supplement: S3 Fig — (TIF) [file pone.0149475.s003.tif]

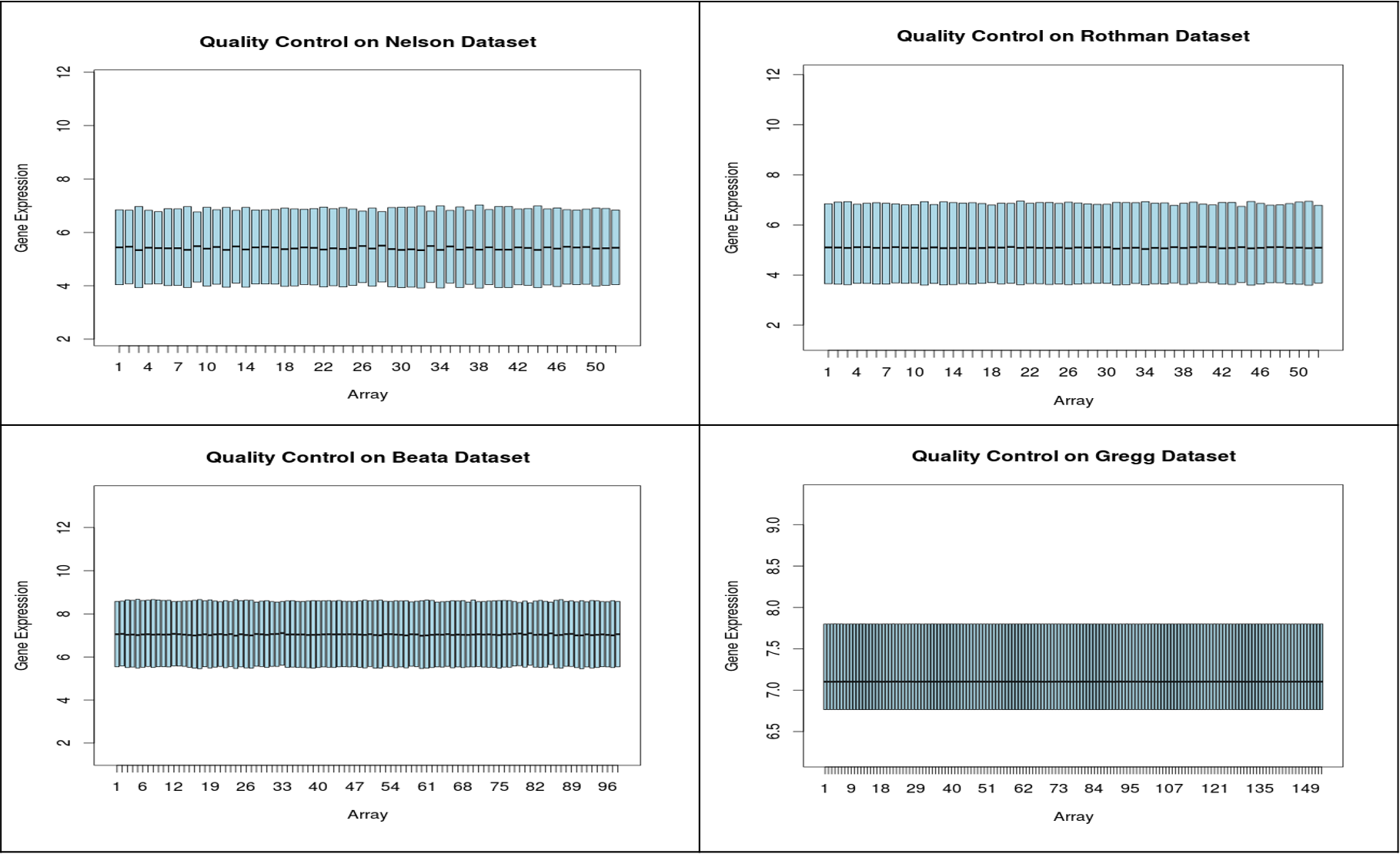

Supplement: S4 Fig — (TIFF) [file pone.0149475.s004.tiff]

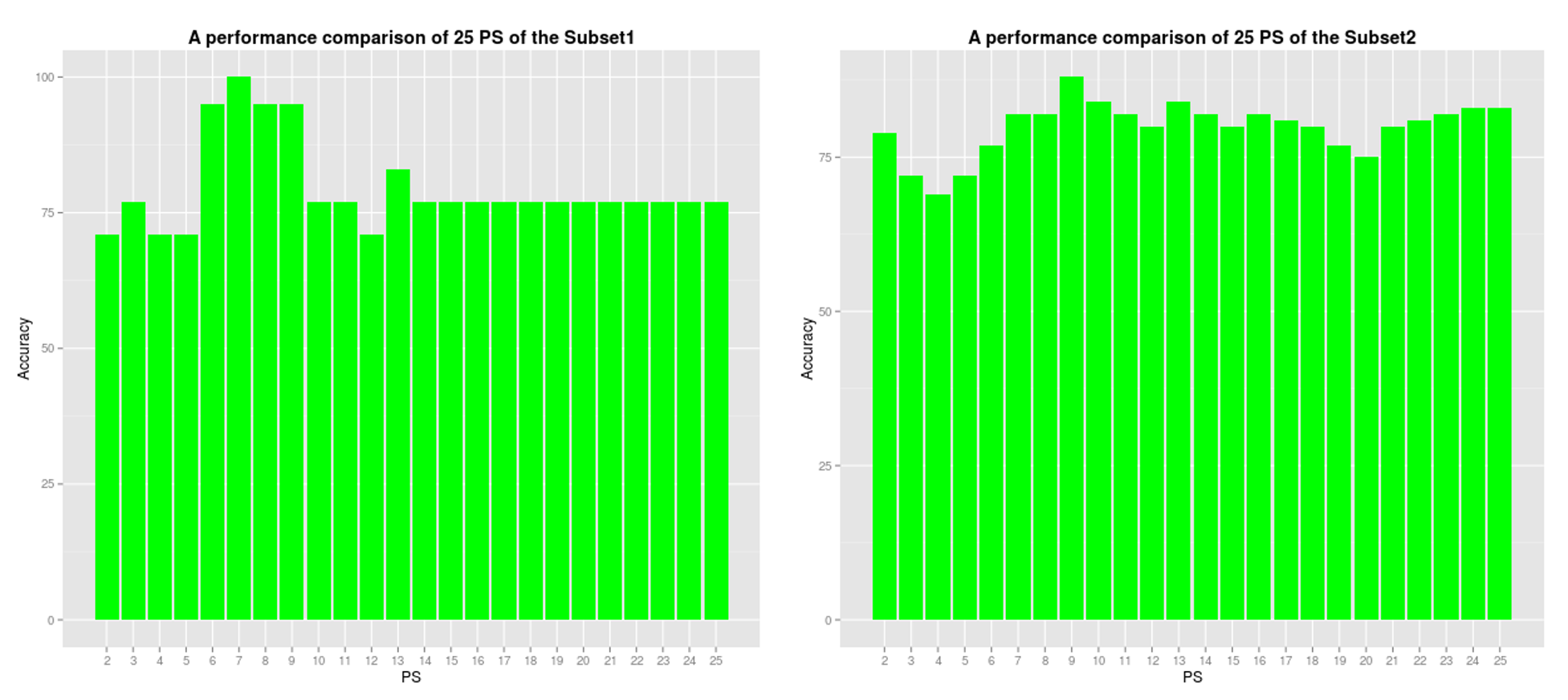

Supplement: S5 Fig — (TIFF) [file pone.0149475.s005.tiff]

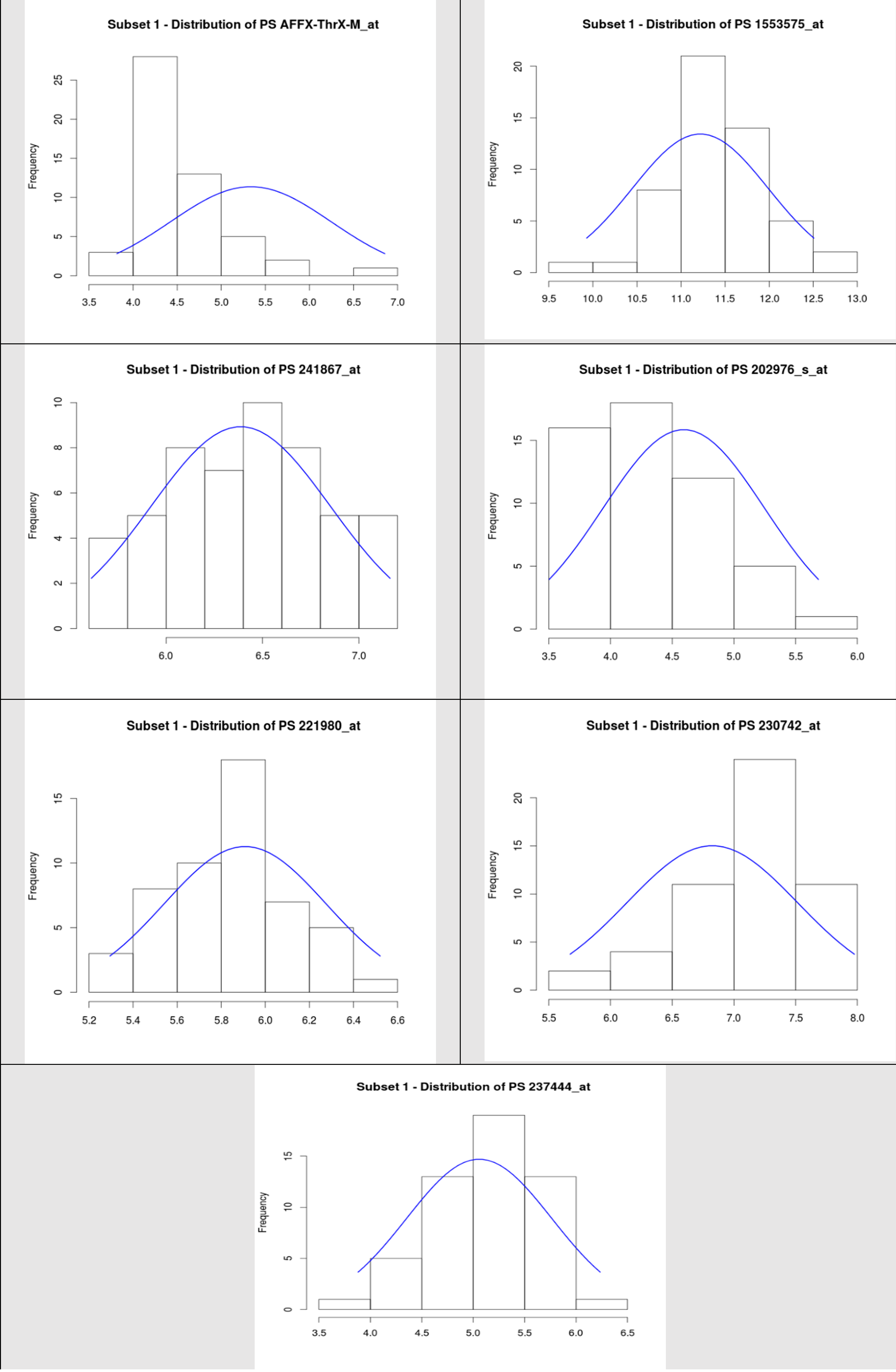

Supplement: S6 Fig — (TIF) [file pone.0149475.s006.tif]

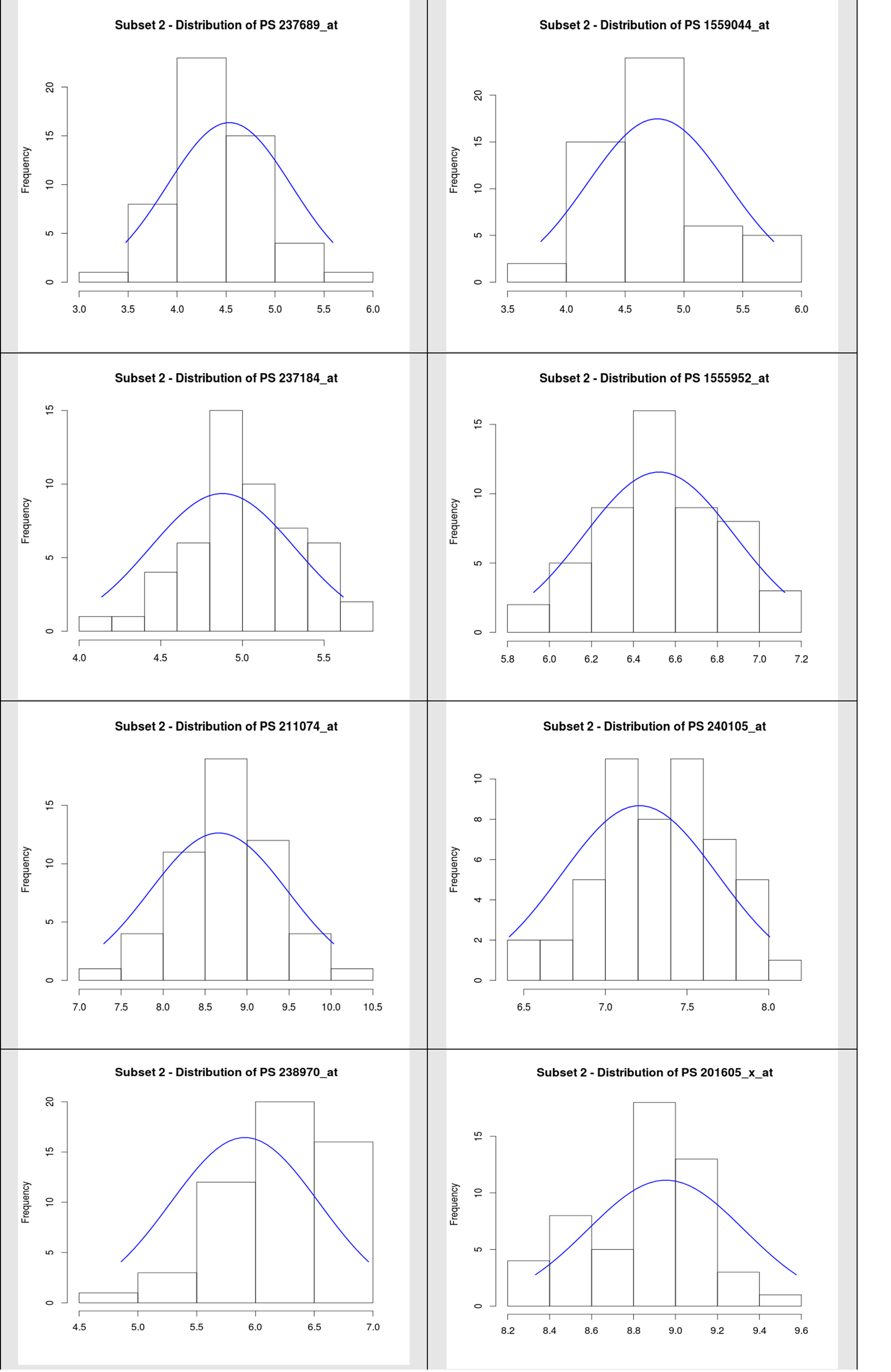

Supplement: S7 Fig — (TIF) [file pone.0149475.s007.tif]

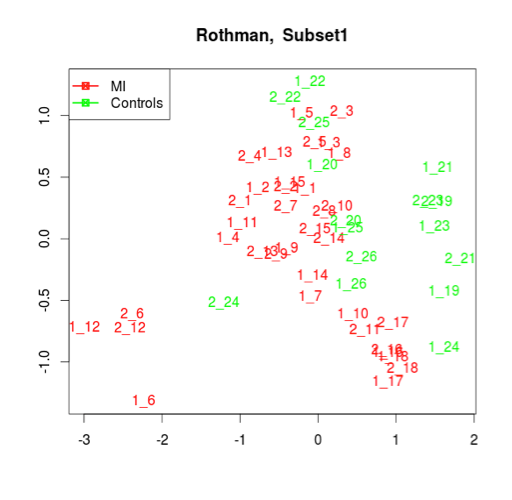

Supplement: S8 Fig — (TIF) [file pone.0149475.s008.tif]
