# Supplementary material for: Diagnosis of Coronary Heart Diseases Using Gene Expression Profiling; Stable Coronary Artery Disease, Cardiac Ischemia with and without Myocardial Necrosis
Source: PLoS One. 2016 Mar 1;11(3):e0149475. doi: 10.1371/journal.pone.0149475 (PMC4773227; doi:10.1371/journal.pone.0149475)
Supplement: S1 File — (PDF) [file pone.0149475.s009.pdf]

The pseudo-code of three optimisation methods are given below.

1. **Optimisation on Success Rate [Optimisation 1]**

```
Initialisation;
currentGene ← 2
geneList ← classifying probesets
while currentGene ≤ totalGenes do
  Perform LOOCV using 1,…,currentGene
  Record SR
  if current SR ≤ previous SR then
     Eliminate currentGene from the geneList
  endif
 currentGene ← currentGene + 1
endwhile
```

2. **Performance Improvement and Random Crossover [Optimisation 2]**

```
Initialisation;
currentGene ← 2
Create initial population
repeat
    while current subset ≤ total subsets do
        Temporary ← current subset
        while currentGene ≤ size of the subset do
            Perform LOOCV using 1,…,currentGene of Temporary
            Record SR and ∆SR
            if current SR ≤  previous SR do
               Eliminate currentGene from Temporary
            endif
            currentGene ← currentGene + 1
        endwhile
        Rank genes of Temporary according to ∆SR
```

```
        Perform LOOCV using top 25 genes of Temporary
        If SR of 25 genes ≥ acceptable do
          Retain the subset
              endif
          endwhile
          Perform 2 point crossover
          Create new population
        until a predefined number of iterations
```

3. **Performance Improvement and Crossover of the Fittest [Optimisation3]**

```
        Initialisation;

currentGene ← 2
Create initial population
repeat
    while current subset ≤ total subsets do
       Temporary ← current subset
        while currentGene ≤ size of the subset do
            Perform LOOCV using 1,…,currentGene of Temporary
            Record SR and ∆SR
            if current SR ≤  previous SR do
              Eliminate currentGene from Temporary
            endif
           currentGene ← currentGene + 1
         endwhile
        Rank genes of Temporary according to ∆SR
        Perform LOOCV using top 25 genes of Temporary
        If SR of 25 genes ≥ acceptable do
          Retain the subset
              endif
          endwhile
```

while subset < total subsets **do**

Rank genes of all subsets according to ΔSR

Perform crossover of top genes

endwhile

Create new population

until a predefined number of iterations
